# Fringe structures and tunable bandgap width of 2D boron nitride nanosheets

**DOI:** 10.3762/bjnano.5.130

**Published:** 2014-07-31

**Authors:** Peter Feng, Muhammad Sajjad, Eric Yiming Li, Hongxin Zhang, Jin Chu, Ali Aldalbahi, Gerardo Morell

**Affiliations:** 1 Institute of Functional Nanomaterials and Department of Physics, College of Natural Sciences, University of Puerto Rico, San Juan, PR/USA 00936-8377; 2 Globalfoundrie, 400 Stone Break Road extension, Malta, NY 12020, USA; 3 Chongqing Institute of Green and Intelligent Technology, CAS, Chongqing 400714, China; 4 King Abdullah Institute for Nanotechnology and Department of Chemistry, KSU, Riyadh 11451, Saudi Arabia

**Keywords:** boron nitride sheets, fringe patterns, functionalization, tunable bandgap width

## Abstract

We report studies of the surface fringe structures and tunable bandgap width of atomic-thin boron nitride nanosheets (BNNSs). BNNSs are synthesized by using digitally controlled pulse deposition techniques. The nanoscale morphologies of BNNSs are characterized by using scanning electron microscope (SEM), and transmission electron microscopy (TEM). In general, the BNNSs appear microscopically flat in the case of low temperature synthesis, whereas at high temperature conditions, it yields various curved structures. Experimental data reveal the evolutions of fringe structures. Functionalization of the BNNSs is completed with hydrogen plasma beam source in order to efficiently control bandgap width. The characterizations are based on Raman scattering spectroscopy, X-ray diffraction (XRD), and FTIR transmittance spectra. Red shifts of spectral lines are clearly visible after the functionalization, indicating the bandgap width of the BNNSs has been changed. However, simple treatments with hydrogen gas do not affect the bandgap width of the BNNSs.

## Introduction

The recent successful investigation of graphene has stimulated interest in atomically thin boron nitride sheets [1–2]. Similar to the method used to produce graphene, BNNSs can be exfoliated from bulk BN crystals by simple mechanical cleavage techniques [3–5]. The problem is that the obtained hBN nanosheets are usually limited by too small size. Therefore, recently most work on synthesis of large BNNSs is based on either chemical-solution-derived method or a chemical vapor deposition (CVD) process. Many excellent results have been reported [6–9]. Systematic and comprehensive reviews of two-dimensional (2D) boron nitride nanostructures: nanosheets, nanoribbons, nanomeshes, and hybrids with graphene have been presented by Lin [10].

Theoretically, surface treatment can effectively control the band gap of nano BN and plays a crucial role of engineering their electrical and electronic properties. For example for BN nanotubes (BNNT), 50% tube surface coverage with chemisorbed hydrogen atoms would cause the BN band gap (which was computed to be 4.29 eV in pristine BNNT) decreased to 2.01 eV [11]. For BNNSs case the adsorption behavior of a single H atom either on the top site of a B or on the top site of an N atom, or two H atoms adsorbed on adjacent B and N sites are also investigated [12]. Using first-principles computations [13] and hybrid density functional theory calculations with van der Waals correction [14], Chen and Zhang show that polar boron nitride (BN) nanoribbons can be favorably aligned via substantial hydrogen bonding at the interfaces, which induces significant interface polarizations and sharply reduces the band gap of insulating BNNSs.

Based on these research, we have experimentally conducted several experiments on using digitally controlled pulse deposition technique to quick synthesis of BNNSs [15] as well as their applications for gas sensors [16] and electronic devices [17–19]. In the present paper, the focus of studies is on variation of the fringe structures and the hydrogen (H) atoms induced band gap width. Chemically shifted components were observed following H treatment, and clear evidence of tunable bandgap width was found.

## Experimental

A pulsed CO_2_ laser deposition technique (CO_2_-PLD: wavelength: 10.6 µm, pulse width: 1–5 µs, repetition rate: 5 Hz, and pulse energy: 5 J) was used. Detailed description of PLD experimental setup can be found in our previous papers [18–19]. Briefly, the laser beam, focused with a 30 cm focal length of ZnSe lens, was incident at 45 degree relative to a rotated (speed of circa 200 rpm) pyrolytic hexagonal BN target (2.00" diameter × 0.125" thick, 99.99% purity, B/N ratio ≈1.05, density ≈1.94 g/ccm) under high vacuum (2.66 × 10^−3^ Pa) chamber. The purpose of the use of the long-focal-length lens is to effectively control the laser-produced plasma beams. The diameter of the focus spot of laser beam on the target was about 2 mm and could be varied by shifting focal lens. The power density of the laser on the target was 2 × 10^8^ W/cm^2^ per pulse. Molybdenum (Mo) and silicon (Si) wafers (1 × 1 cm^2^) as substrates were used and placed 4 cm away from the target. Substrate temperature was controlled by using a thermocouple and heater. Prior to laser irradiation, substrates were rinsed in acetone and methanol in sequence. The duration for each deposition was few minutes. The as-grown samples were then characterized by using SEM, Raman scattering, X-ray diffraction, and FTIR transmittance, respectively. For studies of the nanoscale morphology of BNNSs, the samples were simply scratched off and then transferred to the grids for TEM measurement.

## Results and Discussion

### Fringe structures of boron nitride nanosheets

**Figure 1:**
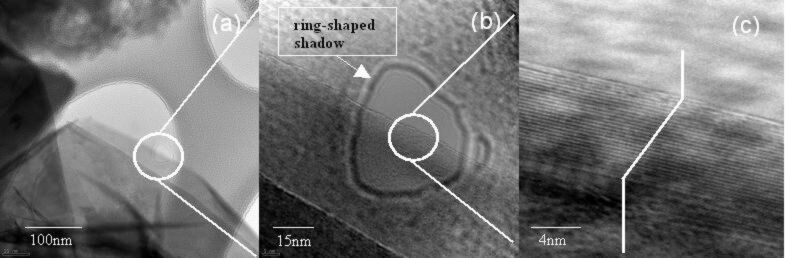
TEM images of BNNSs with different magnifications.

Figure 1 shows TEM images of BNNSs with different magnifications. The sample is prepared at low temperature, around 350 °C. Each as-grown sample normally consists of a large amount of BNNSs that are partially overlapped one another. Average size of each continuous BNNS piece is around a few micrometer squares. The thickness of the BNNS varies from 1 nm to 10 nm. Each BNNS appears highly flat and transparent properties. The well-shaped edge of each BNNS piece is clearly visible as shown in Figure 1a.

Figure 1b shows TEM image with a large magnification, indicating there are many tiny fringes at the edge of the BNNSs. All the fringes have almost the same orientations. Continuing to magnify the TEM image, the highly ordered fringe pattern becomes obvious (Figure 1c), where each fringe is related to a single atomic layer, and thickness of the each atomic layer is around 0.33 nm. Accordingly, the thickness of the obtained BNNS can be estimated around 8 nm (total 25 stacked layers).

It has been noticed that in the most case, low-temperature synthesis yields flat BNNS, whereas high temperature (>500 °C) of synthesis yields relatively high density of intrinsic impurities and the most samples appear curved. A basic reason could be due to the sheets’ stress caused by temperature.

**Figure 2:**
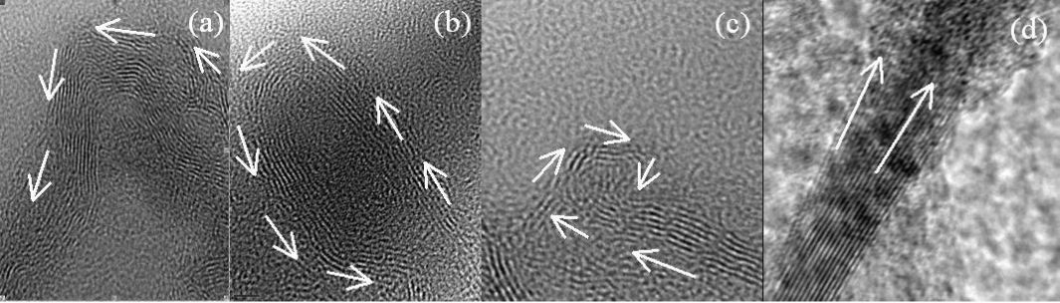
TEM images of BNNSs with a), curved, b), ellipse-shaped bent, c) unusual curled structures, and (d) combination of structures of ripples where tiny ripple construction are related the stacked atomic layers. Width of each ripple/atomic layers is 0.33 nm.

**Figure 3:**
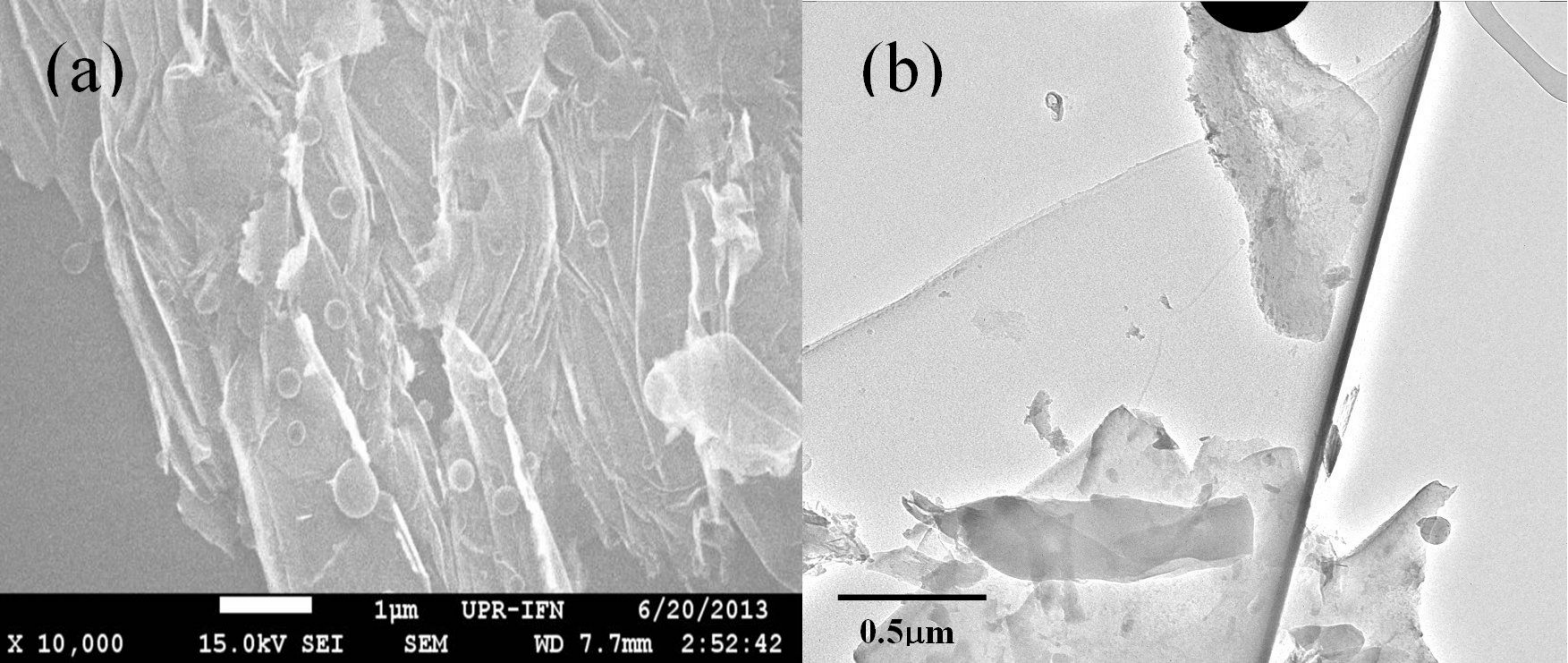
SEM images of BNNSs with (a) curved/wrinkle and (b) folding structure.

With a high-resolution TEM, plenty of rippled structures can be easily observed from the obtained BN samples. Figure 2a shows TEM image of the curved structures. Figure 2b shows TEM image of ellipse-shaped bent structure. Unusual curled structure such as spiraling layer is also observed as shown in Figure 2c that could be related to nanotube in the “parchment model” [20]. Associated with the widths, density or directions of the ripples, in a significant number of cases, we observe various cases, in which two structures of ripples are combined (Figure 2d) because of the random distributions of BNNS pieces. On the other hand, the experimental data above probably suggest that the BNNSs have extremely flexible properties. From SEM images of as-grown BNNSs as shown in Figure 3, ones can easily find that (a) curved/wrinkle and (b) folding structures of BNNSs everywhere. The unique mechanical and electronic properties indicate that the BNNS is a promising material for flexible electronics.

It should be mentioned that a ring-shaped shadow can be identified from the TEM images shown in Figure 1a and 1b. Initially, such shadow is supposed due to the damage of the BNNS. This is because the electron energy used for the electron beam in TEM is usually higher than the knock-on damage threshold [21]. Consequently, the damage of ultra-thin BNNS occurs frequently during TEM measurements in many cases.

**Figure 4:**
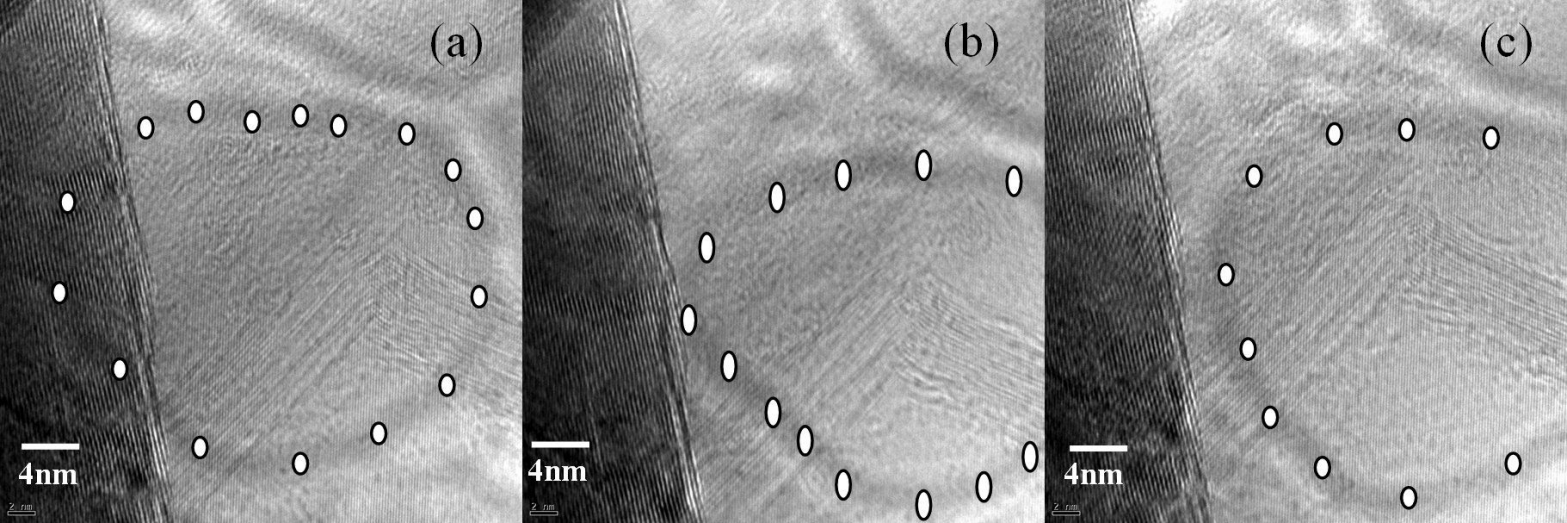
(a–c) TEM images of BNNSs with ring-shaped shadow shifting from the edge. All scale bars are 4 nm.

Interesting is that the shift of the ring-shaped shadow marked with white spots is also observed from the TEM images of BNNS as shown in Figure 4. Normally, any damage of BNNS would be permanent. It seems rare or not possible for the shift of the damaged area. Hence, we expect that the shadows shown in Figure 1 and Figure 4 be rather related to TEM aberrations than to the damage from the high-energy electron beam in the present case.

**Figure 5:**
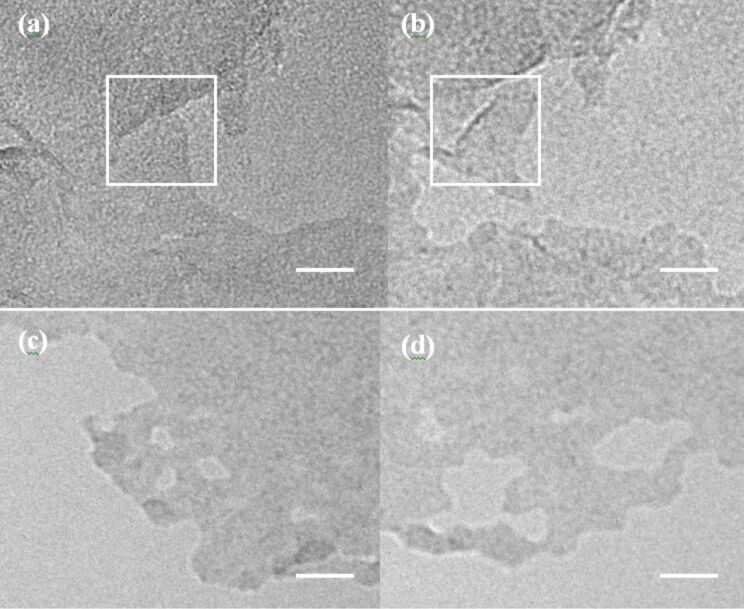
Dynamics in BNNS during TEM measurement a) before and b) after beam. Time between the images is 8 s. Also visible in the sequence is the generation of vacancies within the layer. (c,d) Topological defects are incorporated during this edge reconstruction. On the other edge of the same hole, atoms are removed by the electron beam. Scale bar, 2 nm.

Actually, permanent damage of extremely thin BNNSs occurs frequently at the case of long duration of exposure to high-energy electron beams during TEM measurements. Figure 5a and 5b show the dynamics where the beam damage has just started to form small holes, locally reducing the thickness. After some more irradiation, some “holes” can be found within the irradiated regions (Figure 5c and 5d). Since the product of such “holes” was observed in real time, the process could easily be stopped by switching off the electron beam after formation of the first hole or potentially at the first vacancy through the entire sheet. Figure 5b shows the edge of the atomic thin BNNS. Obviously, the vacancy defect “holes” in the edge area was caused by TEM electron beam. In order to avoid the problems above, super-thin BNNSs are always imaged extremely fast with reduced illumination, or the TEM should be operated at low energy of electron beam.

### Functionalization for tunable bandgap width of BNNSs

**Figure 6:**
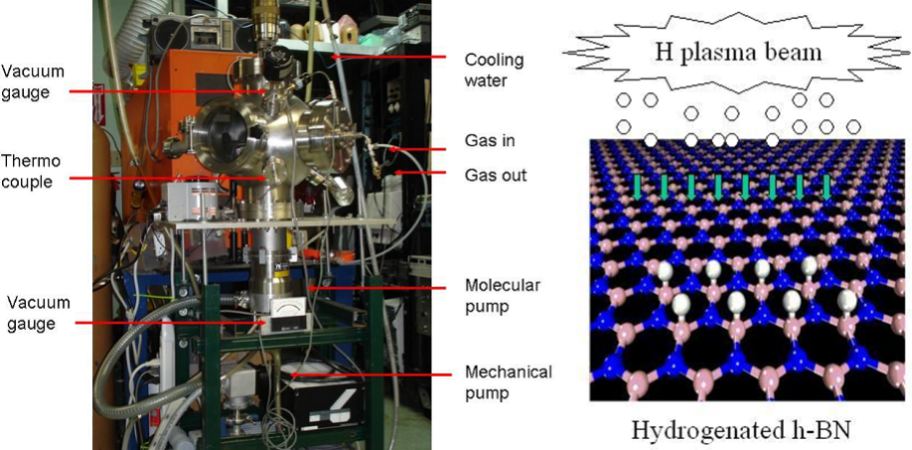
(a) Experimental set up for H plasma functionalization of the BNNSs, and (b) schematic diagram for formation of hydrogenated BNNS.

The wide-band gap of BNNS is a serious obstacle for their application in electronics, despite their high thermal and chemical stabilities. In our previous paper [22], we studied the electric behaviors of hydrogenated BNNS and temperature dependences of resistances before and after hydrogen plasma treatment. The present paper addresses on how to produce atomic BNNS with a desirable bandgap width based on material functionalization. Theoretically, the BNNS band gap would decrease following an increase of atomic hydrogen coverage on the surfaces of BNNSs. Therefore, functionalization of the BNNSs is conducted in a special chamber as shown in Figure 6 based on hydrogen atom plasma beam treatments, and then the samples are characterized using Raman scattering spectroscopy, XRD, and FTIR. From these spectral line profiles and shifts we could investigate variations of the bandgap width and crystalline structures.

**Figure 7:**
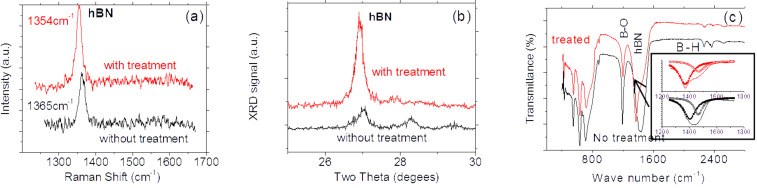
(a) Raman scattering, (b) XRD, and (c) FTIR transmittance spectra of the BNNSs before and after functionalization. Red shifts of all spectral lines is clear visible after functionalization.

Figure 7a shows the Raman spectra of the BNNSs using triple monochromator with an excitation wavelength of 514 nm (Ar^+^ ion Laser). The microscope focused the laser beam onto the surface of the sample. Comparison of two spectral lines before and after treatment, two phenomena can be identified: 1) typical Raman active E_2g_ mode of BNNSs with the hexagonal phase shifts from 1365 cm^−1^ to 1354 cm^−1^. This suggests that the bandgap width of the treated BNNS has been reduced down to 1%, and 2) the width of the spectral line is slightly reduced after the treatment. According to the data from high-resolution TEM and EDX measurements [15,22], we know a pulse deposition technique normally yields high quality of BNNSs where no carbon and other impurities are detected except for a very small amount of oxygen, which is possibly from the residual gas in the chamber. It is expected that the treatment/hydrogenation would cause partial reactions between the hydrogen and oxygen atoms to form water molecules and then evaporation. This process would improve the quality of BNNSs. As a result, the defect concentrations in the treated/hydrogenated BNNSs would decrease, resulting in narrow spectral profile.

Figure 7b shows the XRD pattern of the BNNS sample with and without hydrogenation. The hexagonal structure associated peak shifts from 2θ ≈ 27.02° to 2θ ≈ 26.92° after the treatment. This indicates that the lattice constant of the BNNS hexagonal structure increases up to 1% according to Bragg formulation of X-ray diffraction. Since the bandgap width or energy normally is inversely proportional to the lattice constant for III–V nitride materials [23], we can easily conclude that hydrogen atoms-based functionalization have caused the bandgap width decreased down to 1%. This is in good agreement with the data obtained from the Raman spectrum of BNNSs. It is noticed that a tiny peak positioned at 2θ ≈ 27.8° is related to B_2_O_3_ content present in the original BNNSs without the treatment. As seen in Figure 7b that the B_2_O_3_ content greatly decreases after the hydrogenation.

Figure 7c shows the FTIR spectra of BNNSs in the transmission mode. The dotted (red) and solid (black) spectral lines correspond to the samples with and without the treatment, respectively. A peak recorded at about 1429 cm^−1^ is associated with the in-plane E_1u_ B–N bond stretching vibration of sp^2^-bonded hBN phase [24–25]. It shifts to low wave number down to 1369 cm^−1^ after the treatment, indicating the width of the band gap changes down to 4.3%. This value is quite larger than the results obtained from the Raman and XRD data. The reason is still not clear but to reexamine the FTIP spectra, it can be found that the FTIR spectra actually is dominated by an intense band peaking at 1370.6 cm^−1^ with a shoulder on the higher energy wing at ≈1469 cm^−1^ and the featureless low intensity background at lower wave number. The FTIR peak at ≈1469 cm^−1^ could be assigned as bands bound by impurities or defects, or a phonon replica of bands. The comparison between the normalized FTIR spectra measured with increased spectral resolution from 1200 cm^−1^ to 1800 cm^−1^ for BNNSs before and after functionalization, respectively, is shown in the inset of Figure 7c. It is easy to identify that the hydrogen atom treatments improve the quality of BNNSs. Evident is that the content of the shoulder peak obviously decreases as shown in the inset of Figure 7c. Furthermore, the content of B_2_O_3_ mode at 1200 cm^−1^ is also greatly reduced after the treatment. All these phenomena are now in good agreement with the results obtained from the Raman and XRD measurements. After functionalization, the shoulder peaking remains nearly no shift at ≈1469 cm^−1^, whereas the dominating peak shifts from 1405 to 1370.6 cm^−1^, from which we can conclude the bandgap width has been reduced down to 2.4%.

## Conclusion

Experimental data indicated that low-temperature deposition yields highly flat and transparent BNNSs. The well-shaped edge of BNNS piece is clearly visible, from which the highly ordered fringe patterns can be identified with increased resolution of TEM. Highly ordered fringe structures at the edge are related to atomic layers thickness. In contrast in high-temperature condition, the most samples appear curved. Plenty of unusual curved or curled structures have been observed. These experimental data probably indicate that the BNNSs have extremely flexible mechanic properties.

We conclude that functionalization can be used to realize tunable bandgap width. Red shifts of Raman scattering spectroscopy, XRD, and FTIR transmittance spectral lines confirm that after the treatment, the bandgap width of the BNNSs has been reduced down to 1–2.4%. Furthermore, the treatment/hydrogenation could also cause partial reactions between the hydrogen and oxygen atoms to form water molecules and then evaporation, resulting in decreasing the defect concentrations in treated/hydrogenated BNNSs, and improving the quality of BNNSs.

## References

[1] Dean C R, Young A F, Meric I, Lee C, Wang L, Sorgenfrei S, Watanabe K, Taniguchi T, Kim P, Shepard K L (2010). Nat Nanotechnol.

[2] Lee C, Li Q, Kalb W, Liu X-Z, Berger H, Carpick R W, Hone J (2010). Science.

[3] Lin Y, Williams T V, Connell J W (2010). J Phys Chem Lett.

[4] Du M, Wu Y, Hao X (2013). CrystEngComm.

[5] Wang Y, Shi Z, Yin J (2011). J Mater Chem.

[6] Shi Y, Hamsen C, Jia X, Kim K K, Reina A, Hofmann M, Hsu A L, Zhang K, Li H, Juang Z-Y (2010). Nano Lett.

[7] Yu J, Qin L, Hao Y, Kuang S, Bai X, Chong Y-M, Zhang W, Wang E (2010). ACS Nano.

[8] Song L, Ci L, Lu H, Sorokin P B, Jin C, Ni J, Kvashnin A G, Kvashnin D G, Lou J, Yakobson B I (2010). Nano Lett.

[9] Kim K K, Hsu A, Jia X, Kim S M, Shi Y, Hofmann M, Nezich D, Rodriguez-Nieva J F, Dresselhaus M, Palacios T (2010). Nano Lett.

[10] Lin Y, Connell J W (2012). Nanoscale.

[11] Han S S, Lee S H, Kang J K, Lee H M (2005). Phys Rev B.

[12] Koswattage K R, Shimoyama I, Baba Y, Sekiguchi T, Nakagawa K (2011). J Chem Phys.

[13] Chen W, Li Y, Yu G, Li C-Z, Zhang S B, Zhou Z, Chen Z (2010). J Am Chem Soc.

[14] Zhang Z, Guo W, Yakobson B I (2013). Nanoscale.

[15] Sajjad M, Ahmadi M, Guinel M J-F, Lin Y, Feng P (2013). J Mater Sci.

[16] Sajjad M, Feng P (2014). Mater Res Bull.

[17] Feng P X, Sajjad M (2012). Mater Lett.

[18] Sajjad M, Morell G, Feng P (2013). ACS Appl Mater Interfaces.

[19] Sajjad M, Feng P X (2011). Appl Phys Lett.

[20] Pakdel A, Zhi C, Bando Y, Golberg D (2012). Mater Today.

[21] Zobelli A, Gloter A, Ewels C P, Seifert G, Colliex C (2007). Phys Rev B.

[22] Zhang H X, Feng P X (2012). ACS Appl Mater Interfaces.

[23] Collazo R, Dietz N, Lewerenz H-J, Peter L M (2013). The Group III-Nitride Material Class: from Preparation to Perspectives in Photoelectrocatalysis. Photoelectrochemical Water Splitting.

[24] Paine R T, Narula C K (1990). Chem Rev.

[25] Chen Z-G, Zou J, Liu G, Li F, Wang Y, Wang L, Yuan X-L, Sekiguchi T, Cheng H-M, Lu G Q (2008). ACS Nano.

